# Intradermal delivery of modified mRNA encoding VEGF-A in patients with type 2 diabetes

**DOI:** 10.1038/s41467-019-08852-4

**Published:** 2019-02-20

**Authors:** Li-Ming Gan, Maria Lagerström-Fermér, Leif G. Carlsson, Cecilia Arfvidsson, Ann-Charlotte Egnell, Anna Rudvik, Magnus Kjaer, Anna Collén, James D. Thompson, John Joyal, Ligia Chialda, Thomas Koernicke, Rainard Fuhr, Kenneth R. Chien, Regina Fritsche-Danielson

**Affiliations:** 1 Early Clinical Development, IMED Biotech Unit, AstraZeneca Gothenburg, Pepparedsleden 1, 431 50 Mölndal, Sweden; 2 Department of Molecular and Clinical Medicine, Institute of Medicine, Sahlgrenska Academy at the University of Gothenburg, Arvid Wallgrens backe 1, 413 46 Gothenburg, Sweden; 3 Department of Cardiology, Sahlgrenska University Hospital, Blå stråket 5, 413 45 Gothenburg, Sweden; 4 Cardiovascular, Renal and Metabolism IMED Biotech Unit, AstraZeneca Gothenburg, Pepparedsleden 1, 431 50 Mölndal, Sweden; 5 Moderna, Inc., 200 Technology Square, Cambridge, MA 02139 USA; 6 PAREXEL Early Phase Clinical Unit, Westend Clinic, House 31, 14050 Berlin, Germany; 7 Department of Cell and Molecular Biology, Karolinska Institutet, 171 77 Stockholm, Sweden; 8 Integrated Cardio Metabolic Center, Karolinska Institutet, Blickagången 6, SE-141 57 Huddinge, Sweden

## Abstract

Chemically modified mRNA is an efficient, biocompatible modality for therapeutic protein expression. We report a first-time-in-human study of this modality, aiming to evaluate safety and potential therapeutic effects. Men with type 2 diabetes mellitus (T2DM) received intradermal injections of modified mRNA encoding vascular endothelial growth factor A (VEGF-A) or buffered saline placebo (ethical obligations precluded use of a non-translatable mRNA control) at randomized sites on the forearm. The only causally treatment-related adverse events were mild injection-site reactions. Skin microdialysis revealed elevated VEGF-A protein levels at mRNA-treated sites versus placebo-treated sites from about 4–24 hours post-administration. Enhancements in basal skin blood flow at 4 hours and 7 days post-administration were detected using laser Doppler fluximetry and imaging. Intradermal VEGF-A mRNA was well tolerated and led to local functional VEGF-A protein expression and transient skin blood flow enhancement in men with T2DM. VEGF-A mRNA may have therapeutic potential for regenerative angiogenesis.

## Abstract (web summary)

Chemically modified mRNA is a new approach for therapeutic protein expression that could be applied to angiogenesis. Here the authors show in a phase 1 clinical trial that a modified mRNA encoding VEGF-A is well tolerated in patients with type 2 diabetes.

## Introduction

Therapeutic angiogenesis aims to induce formation of new blood vessels in ischemic tissues by targeted delivery of angiogenic factors and represents a promising approach to the treatment of patients with diseases involving peripheral or myocardial ischemia, such as myocardial infarction, heart failure, stroke, and type 2 diabetes mellitus (T2DM)^1^. The identification of the vascular endothelial growth factor (VEGF) family as powerful mediators of angiogenesis led to human recombinant protein and gene therapy trials, with the most common 165-amino-acid isoform of VEGF-A (VEGF-A_165_) as the leading therapeutic candidate^2,3^. Phase 1/2 clinical trials of VEGF-A gene therapy using DNA plasmid or viral vectors in the 1990s and onward provided valuable safety and tolerability data, but evidence of potential therapeutic benefit for patients with ischemic disease was limited^3–5^.

Chemically modified mRNA is a synthetic and biocompatible modality for therapeutic protein expression^6^. Unlike plasmid DNA and viral gene therapy vectors, modified mRNA offers efficient, dose-dependent, transient protein expression and low innate immunogenicity^6^. For local delivery by direct injection, modified mRNA can be formulated simply in citrate-buffered saline, with no lipid-based carriers^7^. Previous studies have demonstrated the feasibility of mRNA delivery for vaccination in humans^8,9^, but published evidence for therapeutic protein expression and associated improvement in key surrogate disease endpoints following mRNA delivery is limited to data from animal models. In mouse, rat, and pig models of myocardial infarction, intramyocardial injection of modified mRNA encoding VEGF-A_165_ (VEGF-A mRNA) led to elevated cardiac VEGF-A protein levels and improved heart function and survival, which were associated with improved formation of new blood vessels around the infarct^7,10^. Enhanced differentiation of epicardial progenitor cells toward the endothelial lineage was observed when VEGF-A was delivered with mRNA but not when delivered with a DNA plasmid vector^7^. This suggests that the transient and pulse-like expression of VEGF-A from modified mRNA^7^ was essential for increased activation of quiescent progenitor cells following myocardial injury, with resulting formation of functional new blood vessels^7,10^. In contrast, the sustained expression typical of conventional gene therapy vectors leads to formation of angioma-like structures that do not contribute to myocardial blood flow^11^. Damage to small blood vessels in patients with T2DM often leads to inadequate perfusion and impaired wound healing^12–14^. Of patients with T2DM, up to 25% develop a foot ulcer and up to 2% undergo amputation as a result^15,16^, making diabetes the leading cause of non-traumatic amputation in most Western countries^15,16^. When given intradermally in a mouse model, VEGF-A mRNA induced vasodilation, blood flow increase, capillary angiogenesis, and neovascularization^17,18^. In contrast, a non-translatable variant of VEGF-A mRNA lacked vasodilatory and angiogenic activity in mice^17,18^.

Here, we report results from a first-time-in-human study of intradermal VEGF-A mRNA in men with T2DM, aiming to evaluate safety and tolerability as well as VEGF-A protein production and change in skin blood flow. Intradermal VEGF-A mRNA was well tolerated and led to local functional VEGF-A protein expression and transient skin blood flow enhancement in these patients. Our findings indicate that chemically modified mRNA may be a suitable platform for targeted therapeutic protein delivery and suggest that VEGF-A mRNA may potentially induce regenerative angiogenesis.

## Results

### Study design and participants

This was a randomized, double-blind, placebo-controlled, phase 1 study of chemically modified mRNA encoding VEGF-A_165_ (VEGF-A mRNA; AZD8601) in men with T2DM (ClinicalTrials.gov identifier: NCT02935712). The enrolled men with T2DM were aged 41–65 years; all participants were white and most had a body mass index (BMI) above 25 kg/m^2^ (Table 1).

**Table 1:** Baseline characteristics of the enrolled men with T2DM

Study part (*n*): group (*n*)	Part A (*n* = 27): Placebo only^a^ (*n* = 9)	Part A (*n* = 27): VEGF-A mRNA/placebo^a^ (*n* = 18)	Part B (*n* = 15): VEGF-A mRNA/placebo^b^ (*n* = 15)
Age, years			
Median (range)	58.0 (47, 65)	58.5 (41, 65)	56.0 (44, 64)
Race, white			
* n* (%)	9 (100.0)	18 (100.0)	15 (100.0)
BMI, kg/m^2^			
Median (range)	30.80 (27.5–34.5)	29.40 (25.2–33.3)	30.10 (22.2–35.0)
Medical history, *n* (%)			
[>30% in any group]			
Hypertension	5 (55.6)	6 (33.3)	11 (73.3)
Hyperlipidemia	4 (44.4)	5 (27.8)	6 (40.0)
Appendectomy	1 (11.1)	2 (11.1)	8 (53.3)
Inguinal hernia repair	4 (44.4)	3 (16.7)	1 (6.7)
Seasonal allergy	3 (33.3)	3 (16.7)	3 (20.0)

BMI, body mass index; T2DM, type 2 diabetes mellitus; VEGF-A mRNA, vascular endothelial growth factor A modified messenger RNA

^a^VEGF-A mRNA/placebo, placebo/VEGF-A mRNA, or placebo/placebo at sites 1/2

^b^Randomized order of VEGF-A mRNA and placebo injections

The primary objective was to evaluate the safety and tolerability of single ascending doses of VEGF-A mRNA formulated in citrate-buffered saline and given by intradermal injection into the forearm skin, with safety follow-up for 6 months. Pre-specified exploratory objectives were: (1) to evaluate local VEGF-A protein production using microdialysis for 28 h after administration; (2) to compare systemic VEGF-A protein levels after administration with baseline levels; (3) to evaluate the pharmacodynamic effects of VEGF-A mRNA on skin blood flow using laser Doppler fluximetry 4 h after administration; and (4) to evaluate the effects of VEGF-A mRNA on skin blood flow using laser Doppler imaging 7 and 14 days after administration. The study design, including sample size and endpoints, was informed by the findings of an earlier methodological study, in which the feasibility and safety of the methods were also confirmed (Supplementary Note 1).

**Fig. 1:**
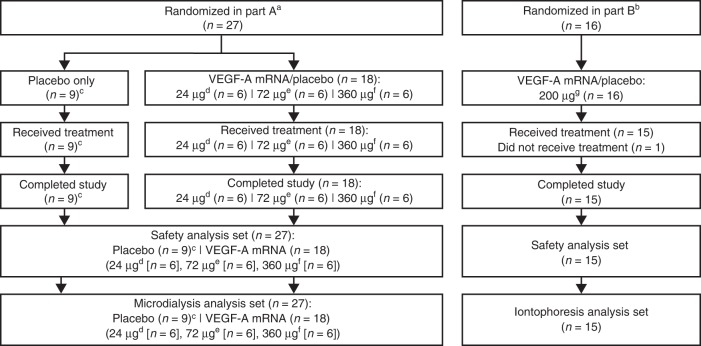
Participant disposition. ^a^Randomization 1:1:1 to VEGF-A mRNA/placebo, placebo/VEGF-A mRNA, or placebo/placebo at injection sites 1/2. ^b^Randomized order of VEGF-A mRNA and placebo at injection sites 1, 2, 3, and 4. ^c^*n* = 3 per cohort (VEGF-A mRNA 24, 72, and 360 µg cohorts). ^d^Given as 6 × 4 µg injections, all at a single site. ^e^Given as 6 × 12 µg injections, all at a single site. ^f^Given as 6 × 60 µg injections, all at a single site. ^g^Given as 2 × 100 µg injections, at different sites. VEGF-A mRNA, vascular endothelial growth factor A modified messenger RNA

The study was divided into part A (single ascending-dose cohorts) and part B (pharmacodynamic cohort) (Fig. 1). In part A, 27 participants were randomized 1:1:1 to receive one of three treatment regimens. Each regimen comprised six 50-µL intradermal injections at one site and six 50-µL injections at a second site on the volar forearm (Supplementary Fig. 1a). Regimens were either VEGF-A mRNA at site 1 and placebo at site 2, placebo at site 1 and VEGF-A mRNA at site 2, or placebo at both sites. Placebo was citrate-buffered saline (the same solution used to formulate VEGF-A mRNA). VEGF-A mRNA doses started at 24 µg per participant (4 µg per injection) in the first cohort of nine participants (VEGF-A mRNA/placebo, *n* = 6; placebo-only, *n* = 3). Doses were increased to 72 µg and 360 µg per participant (12 µg and 60 µg per injection) in two subsequent cohorts, each of nine participants (VEGF-A mRNA/placebo, *n* = 6; placebo/placebo, *n* = 3). In part B, all 15 participants received two 100-µg injections of VEGF-A mRNA (200 µg total dose) and two injections of placebo in a randomized order (Supplementary Fig. 1b). The regimen comprised one 50-µL intradermal injection of either VEGF-A mRNA or placebo at each of the four sites on the volar forearm.

### VEGF-A protein levels

**Fig. 2:**
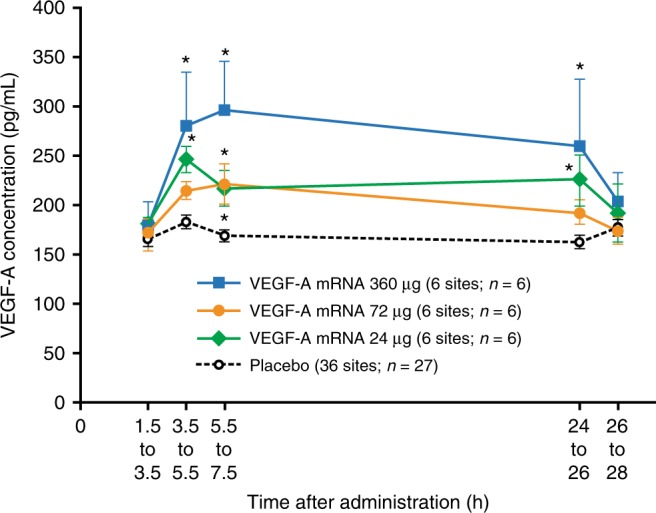
VEGF-A protein levels in microdialysate. Data are mean VEGF-A protein levels from mRNA-treated sites and placebo-treated sites (study part A; *n* = 27). Error bars show SEM (negative error bars omitted from 360 µg dataset for clarity). Placebo dataset includes all placebo injection sites (18 sites in the placebo-only group (*n* = 9; two sites per participant) and 18 sites in the VEGF-A mRNA/placebo group (*n* = 18; one site per participant)). Blue squares, VEGF-A mRNA 360 µg (6 sites; *n* = 6); orange circles, VEGF-A mRNA 72 µg (6 sites; *n* = 6); green diamonds, VEGF-A mRNA 24 µg (6 sites; *n* = 6); black circles, placebo (36 sites: *n* = 27); *p < 0.05 versus combined placebo (ad hoc mixed-effects ANOVA; nominal *p* values). ANOVA, analysis of variance; SEM, standard error of the mean; VEGF-A mRNA, vascular endothelial growth factor A modified messenger RNA

Levels of VEGF-A protein in the injection-site regions in study part A were assessed by skin microdialysis. Mean VEGF-A protein levels in microdialysate from mRNA-treated sites were significantly elevated after administration, compared with mean levels across all placebo sites from all participants (Fig. 2). At each microdialysis sampling time, mean VEGF-A protein levels in microdialysate across all mRNA-treated sites from participants in each cohort were compared with mean levels across all placebo sites from participants in all cohorts. In the 360-µg cohort, mean VEGF-A protein levels at mRNA-treated sites were 38% higher at 3.5–5.5 h (*p* < 0.05), 50% higher at 5.5–7.5 h (*p* < 0.05), and 36% higher at 24–26 h (*p* < 0.05) after administration, compared with placebo-treated sites (ad hoc mixed-effects ANOVA). In the 72-µg cohort, mean VEGF-A protein levels at mRNA-treated sites were 27% higher than at placebo-treated sites at 5.5–7.5 h after administration (*p* < 0.05). In the 24-µg cohort, mean VEGF-A protein levels at mRNA-treated sites were 32% higher at 3.5–5.5 h (*p* < 0.05), 26% higher at 5.5–7.5 h (*p* < 0.05), and 32% higher at 24–26 h (*p* < 0.05) after administration than at placebo-treated sites (ad hoc mixed-effects ANOVA). At 26–28 h after administration, there was no statistically significant increase in VEGF-A protein levels in any of the cohorts compared with placebo-treated sites (Fig. 2).

**Fig. 3:**
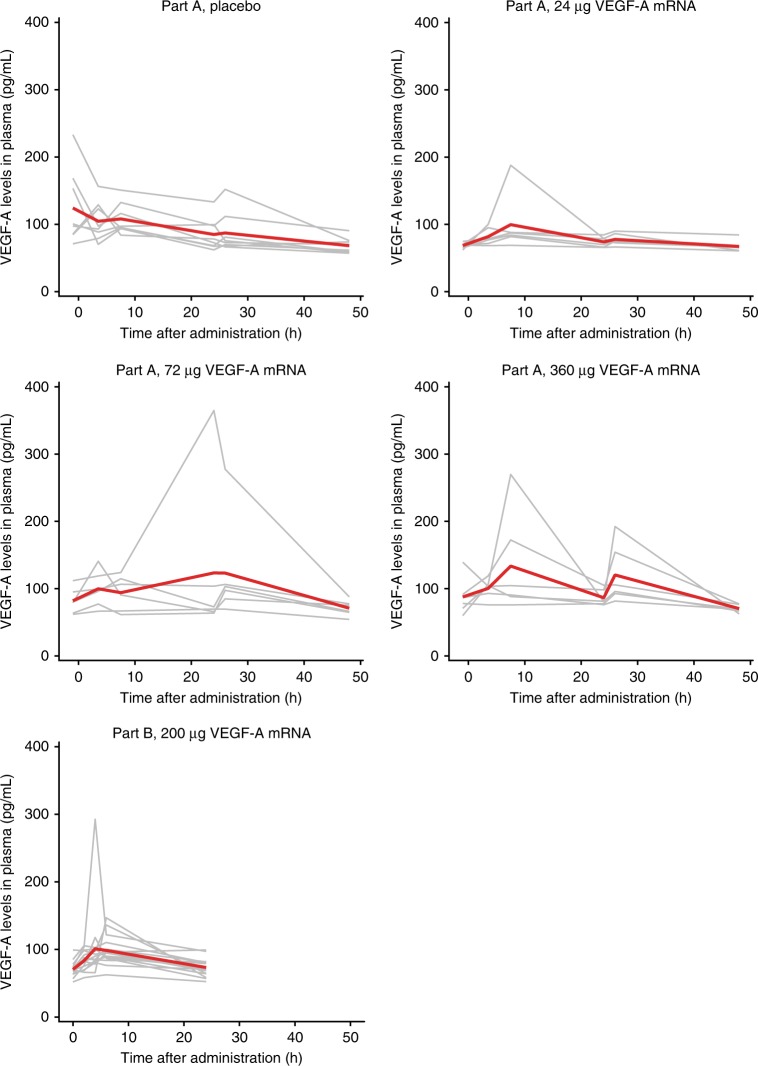
Systemic VEGF-A protein levels. Red lines indicate means and gray lines indicate values in individual participants in study parts A (*n* = 27) and B (*n* = 15). VEGF-A mRNA, vascular endothelial growth factor A modified messenger RNA

Systemic VEGF-A protein concentrations were assessed in parts A and B to confirm that increases in VEGF-A levels following VEGF-A mRNA administration were restricted to the injection-site regions. Mean plasma VEGF-A protein levels remained similar to baseline levels in both parts A and B and were similar in participants receiving VEGF-A mRNA/placebo and placebo only in all dose cohorts in part A (Fig. 3). Transient, isolated plasma VEGF-A elevations in one or two individual participants were observed in all cohorts in parts A and B, including participants in the placebo-only group in part A (Fig. 3).

### Local skin blood flow

**Fig. 4:**
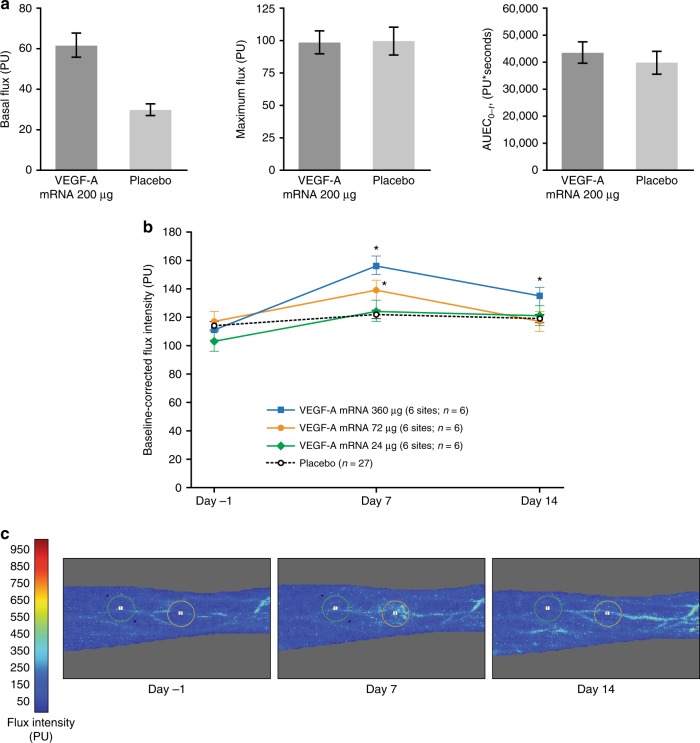
Local skin blood flow outcomes. **a** Laser Doppler fluximetry 4 h after administration at VEGF-A mRNA injection sites and placebo sites (study part B, *n* = 15). Basal flux was measured before acetylcholine iontophoresis, and maximum flux and AUEC_0–*t*_ were measured after acetylcholine iontophoresis. **b** Laser Doppler imaging 7 and 14 days after administration (study part A, *n* = 27). Blue squares, VEGF-A mRNA 360 µg (6 sites; *n* = 6); orange circles, VEGF-A mRNA 72 µg (6 sites; *n* = 6); green diamonds, VEGF-A mRNA 24 µg (6 sites; *n* = 6); black circles, placebo (36 sites; *n* = 27). **c** Representative laser Doppler images showing the two injection sites at each time-point (study part A). Data are means with error bars showing SEM; *p < 0.05, change from baseline (day −1) versus placebo (ad hoc mixed-effects repeated measurements model; nominal *p* values). AUEC_0–*t*_, area under the effect curve from start to end of acetylcholine iontophoresis; PU, perfusion units; SEM, standard error of the mean; VEGF-A mRNA, vascular endothelial growth factor A modified messenger RNA

The effect of mRNA injection on local skin blood flow in part B was assessed using Laser Doppler fluximetry and acetylcholine iontophoresis. Mean basal flux was 2.1-fold higher at VEGF-A mRNA-treated sites than at placebo-treated sites, measured 4 h after administration (Fig. 4a). Mean maximum flux and mean area under the effect curve from the start to the end of acetylcholine iontophoresis (AUEC_0–*t*_) after acetylcholine-induced vasodilation were similar at VEGF-A mRNA-treated sites and placebo-treated sites (Fig. 4a). Iontophoresis had no significant effect on participants’ mean blood pressure and pulse.

Laser Doppler imaging was used in part A to assess changes in skin blood flow up to 14 days after administration. Mean flux intensity at the injection site regions on day 7 after administration was significantly increased with VEGF-A mRNA 360 µg and VEGF-A mRNA 72 µg compared with placebo (Fig. 4b, c; both *p* < 0.05 (ad hoc mixed-effects repeated measurements model)). On day 14, mean flux intensity had declined, but remained significantly increased in participants receiving VEGF-A mRNA 360 µg compared with those receiving placebo (Fig. 4b, c; *p* < 0.05 (ad hoc mixed-effects repeated measurements model)).

### Correlation between blood flow and VEGF-A protein concentration

A linear correlation was observed between the change in flux intensity from baseline to day 7 in part A and both area under the microdialysate VEGF-A protein concentration curve from 3.5 to 28 h (Pearson’s *r* = 0.60, *p* < 0.05; Supplementary Fig. 2a) and maximum microdialysate VEGF-A protein concentration between 3.5 and 28 h (Pearson’s *r* = 0.60, *p* < 0.05; Supplementary Fig. 2b).

### Safety and tolerability

The only causally treatment-related adverse events were injection-site reactions, occurring in 18/18 participants (100%) and 14/15 participants (93%) receiving VEGF-A mRNA in study parts A and B, respectively (Table 2). All adverse events of injection-site reaction were of mild intensity. No adverse events of injection-site reaction were observed at placebo-treated sites (although three participants in the placebo-only group in part A had mild adverse events of injection-site erythema). No deaths, serious adverse events, severe adverse events, or adverse events leading to discontinuation occurred.

**Table 2:** Adverse events

Study part (*n*): group (*n*)	Part A (*n* = 27): Placebo only^a^ (*n* = 9)	Part A (*n* = 27): VEGF-A mRNA/placebo^a^ (*n* = 18)	Part B (*n* = 15): VEGF-A mRNA/placebo^b^ (*n* = 15)
Participants with any AE, *n* (%)	5 (55.6)	18 (100.0)	14 (93.3)
Causally treatment-related, *n* (%)	0	18 (100.0)	14 (93.3)
Treatment unrelated, *n* (%)	5 (55.6)	0	0
Participants with causally treatment-related AEs, *n* (%)			
Injection-site reaction [mild]	0	18 (100.0)	14 (93.3)
Participants with treatment-unrelated AEs, *n* (%)			
Injection-site reaction [mild]	1 (11.1)	0	0
Injection-site erythema [mild]	1 (11.1)	2 (11.1)	0
Asthenia [mild]	0	1 (5.6)	0
Tinea pedis [mild]	0	0	1 (6.7)
Arthropod bite [mild]	0	1 (5.6)	1 (6.7)
Injury [moderate]	0	1 (5.6)	0
Skin abrasion [mild]	0	1 (5.6)	0
Muscle spasms [mild]	0	1 (5.6)	0
Back pain [mild or moderate]	2 (22.2)	0	0
Myalgia [moderate]	0	0	1 (6.7)
Dizziness [mild]	0	1 (5.6)	0
Headache [mild]	1 (11.1)	0	0
Pruritus [mild]	0	1 (5.6)	0
Tooth extraction [mild]	0	1 (5.6)	0
Nasopharyngitis [moderate]	1 (11.1)	0	0

AE, adverse event; VEGF-A mRNA, vascular endothelial growth factor A modified messenger RNA

^a^VEGF-A mRNA/placebo, placebo/VEGF-A mRNA, or placebo/placebo at injection sites 1/2

^b^Randomized order of VEGF-A and placebo injections

At VEGF-A mRNA injection sites, median local reaction summary scores reached or exceeded 2.0 from 6 to 48 h post-administration, but did not exceed 3.5 (on a scale of 0–15); and individual local reaction scores reached at least 1 in all participants but did not exceed 5 in any participant. At placebo injection sites, median local reaction scores did not exceed 1.0 and individual scores did not exceed 2 in any participant.

None of the predefined study stopping criteria were met (Supplementary Table 1) and no clinically concerning safety findings or trends were identified by the investigator while participants were resident at the study center or during the 6-month follow-up period. Some participants’ laboratory parameters shifted to abnormally high or abnormally low values from baseline (Supplementary Table 2) and some had abnormal vital signs during the study (Supplementary Table 3); none of these was considered clinically relevant by the investigator. Changes in laboratory parameters from baseline were generally minor and did not indicate inflammation or alterations in kidney or liver function (Supplementary Dataset 1).

## Discussion

Intradermal injection of VEGF-A mRNA was well tolerated in this randomized, placebo-controlled first-time-in-human study, with mild injection-site reactions the only causally treatment-related adverse events. Exploratory outcomes of the study also suggested that VEGF-A encoded and delivered by chemically modified mRNA was produced in the skin and enhanced skin blood flow in men with T2DM. Local VEGF-A protein levels were elevated from about 4 to 24 h after administration at VEGF-A mRNA-treated sites compared with placebo-treated sites, as assessed by skin microdialysis, with no clinically significant elevation in plasma VEGF-A levels. A twofold enhancement in basal skin blood flow at 4 h after administration was detected using laser Doppler fluximetry, and a sustained enhancement at 7 days after administration was revealed using laser Doppler imaging. To minimize potential risks to the participating volunteers, the only ethically acceptable control treatment in a clinical study of modified mRNA is placebo (in this case, buffered saline “vehicle”). Enhanced skin blood flow on day 7 could therefore be due to an inflammatory reaction or other properties of the mRNA unrelated to VEGF-A protein expression. The finding that non-translatable variants of VEGF-A mRNA lack vasodilatory and angiogenic activity will therefore remain confined to animal models^17,18^. Nevertheless, the results of the present study suggest that this clinically deployable modality for therapeutic VEGF-A delivery may hold promise for the treatment of ischemic diseases.

The only adverse events causally related to VEGF-A mRNA in the present study were mild injection-site reactions. Local reaction scores were higher at VEGF-A mRNA-treated sites than at placebo-treated sites, suggesting that the local reactions at mRNA-treated sites may have resulted from redness due to VEGF-A-mediated vasodilation, with a minor component due to the injection itself or microdialysis membrane insertion at placebo-treated sites. Although VEGF-A mRNA has been shown not to activate the innate immune system in rats and cynomolgus monkeys^10^, the use of citrate-buffered saline as a placebo precluded evaluation of potential non-specific reactogenic effects of the mRNA backbone in the present study. No cardiovascular, laboratory or other findings of concern were noted in the study, and infrequent abnormal values were attributed to T2DM or other factors unrelated to participation in the study.

Microdialysate VEGF-A protein levels at mRNA-treated sites peaked at a mean of about 300 pg/mL from 5.5 to 7.5 h after administration and declined to baseline levels after 26 h, whereas mean levels at placebo-treated sites remained below 185 pg/mL throughout the observation period. These findings are consistent with expression of VEGF-A protein from the administered modified mRNA. To our knowledge, no appropriate data are available for quantitative comparison of the local, transient pulse of VEGF-A protein levels following modified mRNA delivery with endogenous VEGF-A levels or other VEGF-A treatment modalities. The microdialysis methods and VEGF-A assays used in the present studies differed from those used in previous studies, and were based on the findings of the earlier methodological study (Supplementary Note 1). Nevertheless, the twofold increase in basal skin blood flow at 4 h compared with placebo suggests that VEGF-A mRNA treatment was sufficient to induce vasodilation, as detected using laser Doppler fluximetry. The lack of further enhancement of blood flow after acetylcholine iontophoresis suggests vasodilation had reached a maximal limit at all injection sites.

Although VEGF-A protein levels in microdialysate had declined to baseline levels after 26 h, enhanced basal skin blood flow with VEGF-A mRNA treatment was evident on day 7 after administration, as detected using laser Doppler imaging. This safety study was not designed to reveal the mechanisms responsible for this finding, which could include sustained vasodilation, improved endothelial cell function, and capillary angiogenesis. In a mouse model, however, intradermal administration of VEGF-A mRNA did induce neovascularization 7–14 days after injection, as well as vasodilation^17,18^. Furthermore, in animal models of myocardial infarction, VEGF-A mRNA administration to the heart muscle increased the blood vessel density around the infarct and improved cardiac performance at up to 2 months after ligation of a coronary artery^7,10^. Basal skin blood flow at VEGF-A mRNA-treated sites declined between day 7 and day 14 after administration in this study following the increase at day 7. This safety study was not designed to provide any information on the potential mechanisms responsible for these exploratory findings, or to confirm that they require VEGF-A expression from the mRNA. The speculative possibility remains that long-term follow-up treatment with VEGF-A mRNA may be necessary to promote sustained angiogenesis and long-term improvements in blood flow.

Although baseline microvascular function was not directly assessed, it is notable that the volunteers with T2DM had lower baseline flux, maximal flux, and AUEC_0–*t*_ compared with healthy volunteers in the methodological study that preceded the present study (Supplementary Note 1). The 1.8-fold difference in mean basal flux observed between healthy men and those with T2DM at saline-treated sites using laser Doppler fluximetry in the methodological study is similar to the 2.1-fold difference in mean basal flux observed between VEGF-A mRNA-treated and placebo-treated sites in the present study. This suggests that the magnitude of the acute vasodilatory effect of VEGF-A mRNA may lie within a pathophysiologically relevant range, and that basal skin blood flow may have been transiently normalized at VEGF-A mRNA injection sites in men with T2DM following treatment in the present study.

The design of this study had both strengths and limitations. A key strength was the use of pre-specified exploratory endpoints to assess local VEGF-A protein levels and skin blood flow, with sample sizes based on the aforementioned methodological study. VEGF-A mRNA treatment had no possible therapeutic benefit to patients with T2DM in this first-time-in-human safety study. Accordingly, the small number of participants enrolled in the study was considered the smallest required for interpretable results, without placing the participants at unnecessary risk. The study was not designed to demonstrate that VEGF-A expression results solely from translation of the administered mRNA. As described above, ethical considerations preclude the use of a non-translatable variant of VEGF-A mRNA as a control in humans, but this has been tested in preclinical studies and been shown to have no vasodilatory or angiogenic activity in mice^17,18^. The need to use different injection-site patterns for microdialysis and laser Doppler fluximetry outcomes meant that the doses per participant and per site differed between parts A and B of the study, but this did not affect interpretation of the results. Finally, although the exploratory endpoints were pre-specified, statistical analyses of the results were post hoc and *p* values are nominal.

The advantages of chemically modified mRNA as a therapeutic modality include biocompatibility, dose-dependent transient-pulse protein expression, lack of induction of innate immune responses, and large-scale manufacturability^6^. In the present study, delivery of VEGF-A mRNA in buffered saline to human skin was not accompanied by any indications of inflammation in the blood biochemistry and hematology data. Furthermore, local reactions were all mild and may be attributable more to VEGF-A-mediated vasodilation than inflammation. These findings support further clinical investigation of VEGF-A-modified mRNA as an angiogenic therapy not only in patients with peripheral ischemia associated with T2DM but also in those with ischemic cardiovascular diseases. Phase 2 and 3 clinical trials of previous angiogenic gene therapy agents failed to demonstrate any beneficial effects in patients^19^, and data from animal models indicate that the angiogenic response may be disordered and non-functional^11^. Intramyocardial injection of VEGF-A mRNA, however, has been shown to improve heart function and survival in mouse, rat, and pig models of myocardial infarction, with associated neovascularization around the infarct^7,10^. Data from the mouse model also suggest that the transient pulse-like expression of VEGF-A from mRNA may be particularly effective in driving the recruitment of myocardial precursor cells to angiogenic sites, in contrast to DNA or viral gene delivery vectors^7^. A phase 2 placebo-controlled study of epicardially injected VEGF-A mRNA in patients undergoing coronary artery bypass grafting will be conducted in 2018–2019^20^.

In conclusion, although substantial further clinical development is required, this study provides evidence in humans that chemically modified mRNA is well tolerated and may be a suitable platform for targeted therapeutic protein delivery to patients in the clinic. VEGF-A mRNA may hold promise as a regenerative treatment of patients with diabetic wounds and ischemic cardiovascular disease.

## Methods

### Overview and objectives

This was a randomized, double-blind, placebo-controlled, phase 1, first-time-in-human study of modified mRNA encoding VEGF-A_165_ (VEGF-A mRNA; AZD8601) in men with T2DM. The primary objective was to evaluate the safety and tolerability of single ascending doses of VEGF-A mRNA formulated in citrate-buffered saline and given by intradermal injection into the forearm skin. Pre-specified exploratory objectives were: (1) to evaluate local VEGF-A protein production using microdialysis for 28 h after administration; (2) to compare systemic VEGF-A protein levels after administration with baseline levels; (3) to evaluate the pharmacodynamic effects of VEGF-A mRNA on skin blood flow using laser Doppler fluximetry 4 h after administration; and (4) to evaluate the effects of VEGF-A mRNA on skin blood flow using laser Doppler imaging 7 and 14 days after administration. The study was conducted following an earlier methodological study, which was performed to inform the design of the present study and assess the appropriateness of the methods (Supplementary Note 1).

### Conduct and ethics

The study took place between December 2016 and January 2018 at the PAREXEL Early Phase Clinical Unit in Berlin, Germany. The study was conducted in accordance with the principles of the Declaration of Helsinki and the International Conference on Harmonization of Good Clinical Practice. An independent ethics committee/institutional review board (Ethik-Kommission des Landes Berlin, Berlin, Germany) reviewed and approved the study protocol. Once these approvals were obtained, potential participants from the study center's database of patients with diabetes were contacted via phone or letter regarding study participation. Patients wishing to participate were invited to the center to discuss the study with the investigator and to give their informed, written consent before starting the study. The study was registered with ClinicalTrials.gov (identifier: NCT02935712).

### Participants

The study recruited male volunteers with mild T2DM aged 18–65 years and weighing at least 50 kg with a BMI of 20–35 kg/m^2^. T2DM had to be diagnosed at least 1 year before enrollment and treated with no more than two anti-diabetic drugs. Participants had to have stable glycemic control (treatment unchanged for the past 3 months), hemoglobin A1c levels below 10.5%, and fasting plasma glucose levels below or equal to 11.0 mmol/L. Volunteers were excluded if they had: alanine or aspartate aminotransferase levels more than two times the upper limit of normal; hemoglobin levels below 11 g/dL; neutrophil levels below 1500/mm^3^; platelet levels below 100,000/mm^3^; creatinine levels more than 1.2 times the upper limit of normal; or hypertension (resting systolic blood pressure above 150 mmHg or resting diastolic blood pressure above 95 mmHg). Volunteers were also excluded if they had: any recent illness, medical procedure, or trauma; a history of any clinically significant disease or disorder that could affect study participation or results; or any clinically significant laboratory or electrocardiographic abnormality.

### VEGF-A mRNA drug preparation

VEGF-A mRNA drug substance was manufactured in compliance with current Good Manufacturing Practices (GMP) by Moderna, Inc.^8^. Briefly, mRNA was synthesized in vitro using T7 polymerase-mediated transcription from a linearized DNA template containing the VEGF-A open reading frame, flanking 5′ and 3′ untranslated regions and a poly-A tail. A Cap1 structure was enzymatically added to the 5′ end to produce the final mRNA (Supplementary Fig. 3a). Uridine was completely substituted with N1-methylpseudouridine to reduce potential immunostimulatory activity (Supplementary Fig. 3b) and to improve VEGF-A protein expression (Supplementary Fig. 3c) relative to unmodified mRNA. After purification, the mRNA drug substance was diluted in the desired buffer and frozen.

VEGF-A mRNA drug product was prepared at 2 mg/mL for use in the study by dilution of the thawed drug substance solution with a buffer solution containing 2.94 mg/mL sodium citrate dihydrate at pH 6.5 and 7.6 mg/mL sodium chloride, performed at AstraZeneca Gothenburg in compliance with GMP. The final concentration was confirmed by UV assay. The drug product was sterilized by filtration through a 0.22-µm membrane filter and stored in sterilized and depyrogenated vials. A sample for bioburden testing was withdrawn before sterilization (bioburden limit ≤10 CFU/100 mL) and the sterilizing filter was integrity tested. Placebo preparation was performed in a similar manner. All operations requiring sterile conditions were performed within an EU Grade A/US Class 100 processing area.

### Study design

The study was divided into part A (single ascending-dose cohorts) and part B (pharmacodynamic cohort). Local VEGF-A protein levels were measured by microdialysis in part A only, with systemic VEGF-A protein levels assessed in both parts. Skin blood flow was measured by laser Doppler imaging in part A and by laser Doppler fluximetry in part B.

In part A, 27 participants were randomized 1:1:1 to receive one of the three treatment regimens. Each regimen comprised six 50-µL intradermal injections at one site and six 50-µL injections at a second site on the volar forearm (Supplementary Fig. 1a). Regimens were either VEGF-A mRNA at site 1 and placebo at site 2, placebo at site 1 and VEGF-A mRNA at site 2, or placebo at both sites. VEGF-A mRNA doses started at 24 µg per participant (4 µg per injection) in the first cohort of nine participants (VEGF-A mRNA/placebo, *n* = 6; placebo-only, *n* = 3). Doses were increased to 72 µg and 360 µg per participant (12 µg and 60 µg per injection) in two subsequent cohorts, each of nine participants (VEGF-A mRNA/placebo, *n* = 6; placebo/placebo, *n* = 3). In each cohort, three “sentinel” participants were treated first (VEGF-A mRNA, *n* = 2; placebo, *n* = 1) and monitored while resident at the study center from treatment on day 0 until day 7 after treatment. Each participant was treated individually, with 2-day intervals between participants. The remaining six participants in each cohort were then treated, also at 2-day intervals, and monitored while resident from day 0 until day 2 (or for longer if there were safety or tolerability concerns). All participants then attended outpatient follow-up visits on days 14 and 28 and monthly until 6 months after treatment.

In part B, all 15 participants received two VEGF-A mRNA injections and two placebo injections in a randomized order (Supplementary Fig. 1b). The regimen comprised one 50-µL intradermal injection of either VEGF-A mRNA or placebo at each of four sites on the volar forearm. Injections were made very superficially on the volar forearm at least 4 cm apart and at least 5 cm from the wrist and elbow pit. The VEGF-A mRNA dose of 200 µg per participant (100 µg per injection) was selected based on results from part A to provide local VEGF-A protein production and an acceptable safety profile, without exceeding the maximum dose per participant in part A. Each participant was treated individually with 2-day intervals between participants. Participants resided at the study center from treatment on day 0 until at least day 1, then attended outpatient follow-up visits on day 14 and monthly until 6 months after treatment.

### Randomization and blinding details

Participants were randomized within 28 days of screening. Computer-generated randomization sequences were produced by PAREXEL using a protocol supplied by AstraZeneca. In part B, randomization of 15 participants to one of the six possible orders of injection with a block size of six meant that numbers were not fully balanced across treatment orders. VEGF-A mRNA and placebo were matched for appearance, volume, and formulation (citrate-buffered saline). Participants and clinical staff involved in preparing or administering study treatments were blinded to treatment; safety data from each cohort were unblinded for review by the investigator and sponsor.

### Local VEGF-A protein levels in microdialysate (part A)

Approximately 60–90 min after administration of VEGF-A mRNA and/or placebo, a 66 Linear Microdialysis Catheter with a 100-kDa cut-off membrane (MDialysis, Stockholm, Sweden) was inserted ~10 mm intradermally at each of the two injection sites (Supplementary Fig. 1a). Dialysate was collected at 1.5–3.5, 3.5–5.5, and 5.5–7.5 h post-injection using a 107 microdialysis pump (MDialysis) with 0.9% saline solution, and the catheters were then withdrawn. The following day, two new catheters were inserted ~3–5 mm from the previous sites and dialysate was collected at 24–26 and 26–28 h post-injection (Supplementary Fig. 1a). The primary microdialysis endpoint was VEGF-A protein level in the dialysate at each collection time. Measured VEGF-A protein levels were corrected for the dilution factor associated with sample processing. Microdialysate samples with very low volumes were excluded from analysis (volume <20% of the expected volume based on microdialysis flow rate).

### VEGF-A protein levels in plasma (parts A and B)

Plasma samples were taken for analysis of VEGF-A protein concentration before administration and at 3.5, 7.5, 24, 26, and 48 h after administration in part A, and before administration and at 2, 4, 6, and 24 h after administration in part B.

### Skin blood flow by laser Doppler fluximetry (part B)

Four hours after administration of VEGF-A mRNA and/or placebo, skin blood flow at all injection sites was measured using laser Doppler fluximetry (PeriFlux System 5000 with 481 probe, Perimed, Datavägen, Sweden). After measurement of basal perfusion for ~10 min, 1% acetylcholine chloride (Bausch and Lomb GmbH, Berlin, Germany) in water was delivered to the injection sites for ~10 min via constant-current iontophoresis at 10 µA (PF 751 PeriIont USB Power Supply with 383 chamber, Perimed). Fluximetry endpoints were basal flux (before acetylcholine iontophoresis), maximum flux (after acetylcholine iontophoresis), and AUEC_0–*t*_, expressed in arbitrary perfusion units. Blood pressure and pulse rate were monitored during fluximetry, in addition to other safety assessments.

### Skin blood flow by laser Doppler imaging (part A)

Images of skin blood flow in the forearm of each participant were obtained using a moorLDI2 continuous-scanning laser Doppler imager (Moor Instruments, Axminster, UK) at pretreatment baseline (day –1), at 10 h after administration on day 0, and on days 7 and 14 after administration. The area imaged included the entire forearm from 2 cm below the elbow to the wrist, with the images obtained on day 0 used to locate the injection sites in the images from the other study days. Flux intensity in the injection site regions was calculated and corrected for baseline values.

### Safety and tolerability outcomes

Adverse events were monitored from screening until the last follow-up visit. Local reactions were assessed on a summary scale of 0–15 as the sum of scores on investigator-rated scales of 0–3 for redness, bruising, and swelling, and participant-rated scales of 0–3 for itching and pain. Summary scores of 2 or above were reported as an adverse event. Other safety outcomes included monitoring of vital signs (pulse and blood pressure), electrocardiography, physical examinations, and laboratory assessments (hematology, blood biochemistry, coagulation, urinalysis, viral serology, and urine drug and alcohol tests). Predefined study stopping criteria were based on adverse events, cardiovascular findings, and laboratory findings (Supplementary Table 1).

### Sample size

The sample size for this exploratory study was not based on a formal power calculation. Sample size was based on experience from previous studies to minimize exposure of volunteers to study procedures whilst enabling the objectives to be met. A previous methodological study (Supplementary Note 1) evaluated the inter- and intra-individual variability in microdialysate VEGF-A protein levels and laser Doppler fluximetry parameters in healthy volunteers and patients with T2DM following placebo treatment.

### Analysis sets

The safety analysis set included all participants who received VEGF-A mRNA or placebo with available post-administration safety data. The microdialysis analysis set included all participants who received VEGF-A mRNA or placebo with at least one evaluable VEGF-A protein level datum. The iontophoresis analysis set included all participants who received VEGF-A mRNA or placebo with calculable uncorrected AUEC_0–*t*_ for at least one site. Participants with protocol deviations that could have influenced microdialysis or fluximetry outcomes were excluded from the respective analysis set.

### Statistical tests

No inferential hypothesis testing was pre-specified in this exploratory study, with the exception of three paired *t*-tests comparing pulse rate and systolic and diastolic blood pressure before and after acetylcholine iontophoresis (*α* = 0.05). All other statistical analyses were post hoc and *p* values are nominal. All tests were two-sided. For microdialysis outcomes, corrected VEGF-A protein levels at mRNA injection sites in each cohort and at placebo injection sites across all cohorts were compared using log-transformed data in a mixed-effects analysis of variance model with treatment as a fixed effect and participant as a random effect (*α* = 0.05; compound symmetry covariance structure). For laser Doppler microscopy outcomes, change from baseline flux intensity in each cohort was compared with placebo using a mixed-effects repeated measurements model, with treatment, time, and treatment × time as fixed effects, participant as a random effect and baseline as covariate (*α* = 0.05; compound symmetry covariance structure). Pearson's correlation coefficients were calculated for the relationship between change in flux intensity from baseline to day 7 and both the area under the VEGF-A microdialysate protein concentration curve from 3.5 to 28 h and the maximum VEGF-A microdialysate protein concentration between 3.5 and 28 h. One measurement from each participant was included in the analysis. For participants with both VEGF-A mRNA- and placebo-treated sites, the VEGF-A mRNA-treated site was used. For participants with two placebo-treated sites, site 2 was used (see Supplementary Fig. 1a). All statistical analyses were performed using SAS version 9.4.

### Reporting summary

Further information on experimental design is available in the Nature Research Reporting Summary linked to this article.

## Supplementary information

Reporting Summary

Supplementary Information

Peer Review File

Supplementary Data 1

Description of Additional Supplementary Files

Source Data

## Data Availability

This study is registered with ClinicalTrials.gov (identifier: NCT02935712) and is posted on the AstraZeneca Clinical Trials Website (identifier: D9150C00001). The clinical data underlying the findings described in this manuscript may be obtained in accordance with AstraZeneca’s data sharing policy described at https://astrazenecagrouptrials.pharmacm.com/ST/Submission/Disclosure. The nonclinical data underlying Supplementary Fig. 3b, c are provided in the Source Data file.
