# Supplementary material for: Intradermal delivery of modified mRNA encoding VEGF-A in patients with type 2 diabetes
Source: Nat Commun. 2019 Feb 20;10:871. doi: 10.1038/s41467-019-08852-4 (PMC6382754; doi:10.1038/s41467-019-08852-4)
Supplement: Supplementary file 1 — Reporting Summary [file 41467_2019_8852_MOESM1_ESM.pdf]

nature research

Corresponding author(s): Li-Ming Gan

Last updated by author(s): Dec 21, 2018

Reporting Summary

Nature Research wishes to improve the reproducibility of the work that we publish. This form provides structure for consistency and transparency in reporting. For further information on Nature Research policies, see Authors & Referees and the Editorial Policy Checklist.

Statistics

For all statistical analyses, confirm that the following items are present in the figure legend, table legend, main text, or Methods section.

n/a	Confirmed	
☐	☒	The exact sample size (*n*) for each experimental group/condition, given as a discrete number and unit of measurement
☐	☒	A statement on whether measurements were taken from distinct samples or whether the same sample was measured repeatedly
☐	☒	The statistical test(s) used AND whether they are one- or two-sided<br>*Only common tests should be described solely by name; describe more complex techniques in the Methods section.*
☐	☒	A description of all covariates tested
☐	☒	A description of any assumptions or corrections, such as tests of normality and adjustment for multiple comparisons
☐	☒	A full description of the statistical parameters including central tendency (e.g. means) or other basic estimates (e.g. regression coefficient) AND variation (e.g. standard deviation) or associated estimates of uncertainty (e.g. confidence intervals)
☐	☒	For null hypothesis testing, the test statistic (e.g. *F*, *t*, *r*) with confidence intervals, effect sizes, degrees of freedom and *P* value noted<br>*Give P values as exact values whenever suitable.*
☒	☐	For Bayesian analysis, information on the choice of priors and Markov chain Monte Carlo settings
☒	☐	For hierarchical and complex designs, identification of the appropriate level for tests and full reporting of outcomes
☐	☒	Estimates of effect sizes (e.g. Cohen's *d*, Pearson's *r*), indicating how they were calculated

Our web collection on statistics for biologists contains articles on many of the points above.

Software and code

Policy information about availability of computer code

Data collection	The randomization list was generated using RNGEN2 version 1.0.1 (a PAREXEL in-house tool). Data were coded using the iVal database system version 2.7 and collected in ClinBase version 4.6.07. Laser Doppler fluximetry/imaging data were collected using PeriFlux PeriSoft for Windows (PSW) version 2.5.5.
Data analysis	Statistical analyses and table, figure and listing generation were performed using SAS version 9.4.

For manuscripts utilizing custom algorithms or software that are central to the research but not yet described in published literature, software must be made available to editors/reviewers. We strongly encourage code deposition in a community repository (e.g. GitHub). See the Nature Research guidelines for submitting code & software for further information.

Data

Policy information about availability of data

All manuscripts must include a data availability statement. This statement should provide the following information, where applicable:

- Accession codes, unique identifiers, or web links for publicly available datasets
- A list of figures that have associated raw data
- A description of any restrictions on data availability

This study is registered with ClinicalTrials.gov (identifier: NCT02935712) and is posted on the AstraZeneca Clinical Trials Website (identifier: D9150C00001). Data underlying the findings described in this manuscript may be obtained in accordance with AstraZeneca's data sharing policy described at https://astrazenecagrouptrials.pharmacm.com/ST/Submission/Disclosure.

Field-specific reporting

Please select the one below that is the best fit for your research. If you are not sure, read the appropriate sections before making your selection.

☒ Life sciences ☐ Behavioural & social sciences ☐ Ecological, evolutionary & environmental sciences

For a reference copy of the document with all sections, see nature.com/documents/nr-reporting-summary-flat.pdf

Life sciences study design

All studies must disclose on these points even when the disclosure is negative.

Sample size	The sample size for this exploratory study was not based on a formal power calculation. Sample size was based on experience from previous studies to minimize exposure of volunteers to study procedures whilst enabling the objectives to be met. A previous methodological study (Supplementary Note 1) evaluated the inter- and intra-individual variability in microdialysate VEGF-A protein levels and laser Doppler fluximetry parameters in healthy volunteers and patients with T2DM following placebo treatment.
Data exclusions	The safety analysis set included all participants who received VEGF-A mRNA or placebo with available post-administration safety data. The microdialysis analysis set included all participants who received VEGF-A mRNA or placebo with at least one evaluable VEGF-A protein level datum. The iontophoresis analysis set included all participants who received VEGF-A mRNA or placebo with calculable uncorrected AUEC(0–t) for at least one injection site.<br><br>Participants with protocol deviations that could have influenced microdialysis or fluximetry outcomes were excluded from the respective analysis set. One patient in study part A had protocol deviations on day 7, missing laser Doppler imaging/fluximetry measurements for both sites (placebo and VEGF-A mRNA 24 µg), and during follow-up, which was performed 20 days post-dose instead of 14 days post-dose. The patient was excluded for those specific time points but included in the analysis population.
Replication	In both parts of the study, microdialysis and fluximetry outcomes were assessed after injection of VEGF-A mRNA and placebo at multiple forearm sites per participant and in multiple participants. The sample size was based on experience from previous studies, including the aforementioned methodological study (Supplementary Note 1), to minimize exposure of participants to study procedures whilst enabling the objectives to be met. All attempts at replication were successful and statistical tests were performed as described in the methods to assess effect sizes and variation in the data collected for each treatment group and thus estimate the reproducibility of the findings.
Randomization	Participants were randomized within 28 days of screening. Computer-generated randomization sequences were produced by PAREXEL using a protocol supplied by AstraZeneca. In part B, randomization of 15 participants to one of the six possible orders of injection with a block size of six meant that numbers were not fully balanced across treatment orders. VEGF-A mRNA and placebo were matched for appearance, volume and formulation (citrate-buffered saline).
Blinding	VEGF-A mRNA and placebo were matched for appearance, volume and formulation (citrate-buffered saline). Participants and clinical staff involved in preparing or administering study treatments were blind to treatment; safety data from each cohort was unblinded for review by the investigator and sponsor.

Reporting for specific materials, systems and methods

We require information from authors about some types of materials, experimental systems and methods used in many studies. Here, indicate whether each material, system or method listed is relevant to your study. If you are not sure if a list item applies to your research, read the appropriate section before selecting a response.

Materials & experimental systems

n/a	Involved in the study	
☐	☒	Antibodies
☐	☒	Eukaryotic cell lines
☒	☐	Palaeontology
☒	☐	Animals and other organisms
☐	☒	Human research participants
☐	☒	Clinical data

Methods

n/a	Involved in the study	
☒	☐	ChIP-seq
☒	☐	Flow cytometry
☒	☐	MRI-based neuroimaging

Antibodies

Antibodies used	Human VEGF ELISA kit, catalog# DVE00, R&D Systems; Human interferon-beta ELISA kit, catalog# 41410-1, PBL Assay Science.
Validation	See vendor's website.

Eukaryotic cell lines

Policy information about cell lines

Cell line source(s)	HeLa cells, catalog# CCL-2, American Type Culture Collection (ATCC); BJ fibroblasts, catalog# CRL-2522, American Type Culture Collection (ATCC).
Authentication	See vendor's website.
Mycoplasma contamination	Cells were negative for mycoplasma.
Commonly misidentified lines (See ICLAC register)	*Name any commonly misidentified cell lines used in the study and provide a rationale for their use.*

Human research participants

Policy information about studies involving human research participants

Population characteristics	The study recruited male volunteers with mild T2DM aged 18–65 years and weighing at least 50 kg with a body mass index (BMI) of 20–35 kg/m2. T2DM had to be diagnosed at least 1 year before enrolment and treated with no more than two anti-diabetic drugs. Participants had to have stable glycemic control (treatment unchanged for the past 3 months), hemoglobin A1c levels below 10.5% and fasting plasma glucose levels below or equal to 11.0 mmol/L. Volunteers were excluded if they had: alanine or aspartate aminotransferase levels more than two times the upper limit of normal; hemoglobin levels below 11 g/dL; neutrophil levels below 1500/mm3; platelet levels below 100 000/mm3; creatinine levels more than 1.2 times the upper limit of normal; or hypertension (resting systolic blood pressure above 150 mmHg or resting diastolic blood pressure above 95 mmHg). Volunteers were also excluded if they had: any recent illness, medical procedure or trauma; a history of any clinically significant disease or disorder that could affect study participation or results; or any clinically significant laboratory or electrocardiographic abnormality.
Recruitment	Once etichal approvals were obtained, potential participants from the study center´s database of patients with diabetes were contacted via phone or letter regarding study participation. Patients wishing to participate were invited to the center to discuss the study with the investigator and to give their informed, written consent before starting the study.
Ethics oversight	The study was conducted in accordance with the principles of the Declaration of Helsinki and the International Conference on Harmonization of Good Clinical Practice. An independent ethics committee/institutional review board (Ethik-Kommission des Landes Berlin, Berlin, Germany) reviewed and approved the study protocol.

Note that full information on the approval of the study protocol must also be provided in the manuscript.

Clinical data

Policy information about clinical studies

All manuscripts should comply with the ICMJE guidelines for publication of clinical research and a completed CONSORT checklist must be included with all submissions.

Clinical trial registration	NCT02935712
Study protocol	Data underlying the findings described in this manuscript may be obtained in accordance with AstraZeneca's data sharing policy described at https://astrazenecagrouptrials.pharmacm.com/ST/Submission/Disclosure.
Data collection	The study took place between December 2016 and January 2018 at the PAREXEL Early Phase Clinical Unit in Berlin, Germany.
Outcomes	Pre-specified exploratory objectives were: (1) to evaluate local VEGF-A protein production using microdialysis for 28 hours after administration; (2) to compare systemic VEGF-A protein levels after administration with baseline levels; (3) to evaluate the pharmacodynamic effects of VEGF-A mRNA on skin blood flow using laser Doppler fluximetry 4 hours after administration; and (4) to evaluate the effects of VEGF-A mRNA on skin blood flow using laser Doppler imaging 7 and 14 days after administration.
