# Supplementary material for: Intradermal delivery of modified mRNA encoding VEGF-A in patients with type 2 diabetes
Source: Nat Commun. 2019 Feb 20;10:871. doi: 10.1038/s41467-019-08852-4 (PMC6382754; doi:10.1038/s41467-019-08852-4)
Supplement: Supplementary file 2 — Supplementary Information [file 41467_2019_8852_MOESM2_ESM.pdf]

SUPPLEMENTARY INFORMATION

Intradermal delivery of modified mRNA encoding VEGF-A in patients with type 2 diabetes

Li-Ming Gan[1,2,3,*], Maria Lagerström-Fermér[1], Leif G Carlsson[4], Cecilia Arfvidsson[1], Ann-Charlotte Egnell[1], Anna Rudvik[1], Magnus Kjaer[1], Anna Collén[4], James D Thompson[5], John Joyal[5], Ligia Chialda[6], Thomas Koernicke[6], Rainard Fuhr[6], Kenneth R Chien[7,8], Regina Fritsche-Danielson[4]

[1]Early Clinical Development, IMED Biotech Unit, AstraZeneca Gothenburg, Pepparedsleden 1, 431 50 Mölndal, Sweden
[2]Department of Molecular and Clinical Medicine, Institute of Medicine, Sahlgrenska Academy at the University of Gothenburg, Arvid Wallgrens backe 1, 413 46 Gothenburg, Sweden
[3]Department of Cardiology, Sahlgrenska University Hospital, Blå stråket 5, 413 45 Gothenburg, Sweden
[4]Cardiovascular, Renal and Metabolism IMED Biotech Unit, AstraZeneca Gothenburg, Pepparedsleden 1, 431 50 Mölndal, Sweden
[5]Moderna, Inc., 200 Technology Square, Cambridge, MA 02139, USA
[6]PAREXEL Early Phase Clinical Unit, Westend Clinic, House 31, 14050 Berlin, Germany
[7]Department of Cell and Molecular Biology, Karolinska Institutet, 171 77 Stockholm, Sweden
[8]Integrated Cardio Metabolic Center, Karolinska Institutet, Blickagången 6, SE-141 57 Huddinge, Sweden

*Corresponding author: Professor Li-Ming Gan MD PhD, IMED Biotech Unit, AstraZeneca Gothenburg, Pepparedsleden 1, 431 50 Mölndal, Sweden | +46 (31) 776 1209 | li-ming.gan@astrazeneca.com

Contents

Supplementary Notes.. 3

Supplementary Note 1. Methodological Validation Study.. 3

Supplementary Figures 13

Supplementary Figure 1. Injection sites 13

Supplementary Figure 2. Correlations of local skin blood flow with VEGF-A levels.......... 14

Supplementary Figure 3. VEGF-A mRNA characteristics 15

Supplementary Tables 17

Supplementary Table 1. Study stopping criteria... 17

Supplementary Table 2. Numbers of participants whose laboratory values shifted to abnormally low or high values from other baseline values 18

Supplementary Table 3. Participants with at least one abnormal vital sign reading during the study.. 20

Supplementary Notes

Supplementary Note 1. Methodological Validation Study

INTRODUCTION

This methodological study was performed in advance of the phase 1 study of AZD8601 VEGF-A mRNA to evaluate the safety and technical feasibility of the microdialysis and laser Doppler fluximetry assessments, as well as to inform sample size and outcome measure selection. The methodological study investigated microdialysis for assessment of local VEGF-A protein concentrations and laser Doppler fluximetry for assessment of forearm skin blood flow in healthy volunteers and patients with mild and well-controlled type 2 diabetes mellitus (T2DM).

METHODS

Objectives

The primary objectives of the microdialysis section of the study were to evaluate the safety and tolerability of the microdialysis procedure, and to establish baseline values and the inter- and intra-individual variability of VEGF-A protein concentrations in microdialysate. The effect of local saline injection on microdialysis outcomes was compared with untreated sites, as AZD8601 was to be administered in saline in the subsequent phase 1 study.

The primary objective of the laser Doppler fluximetry section of the study was to assess the feasibility of the procedure for measurement of skin blood flow, which included evaluation of intra- and inter-individual variability of baseline and acetylcholine-induced vasodilation in forearm skin. The effect of local saline injection on the laser Doppler fluximetry measurements was compared with untreated sites.

Exploratory objectives included investigation of the potential differences between healthy volunteers and T2DM patients regarding local VEGF-A production in dialysate and skin blood flow response during acetylcholine challenge.

Safety assessments comprised monitoring of adverse events and vital signs (pulse and blood pressure), as well as electrocardiography, physical examinations, and laboratory assessments (hematology, clinical chemistry, urinalysis, viral serology, and urine drug and alcohol tests).

Study conduct and design

The study was performed at a single study center, the PAREXEL Early Phase Clinical Unit in Berlin, Germany, between March 2016 and May 2016. The study was conducted in accordance with the principles of the Declaration of Helsinki and the International Conference on Harmonisation of Good Clinical Practice. An independent ethics committee and institutional review board reviewed and approved the study protocol. All participants freely gave their informed, written consent before starting the study.

The study comprised a screening period of up to 21 days followed by an experimental period during which participants were resident from the morning of day −1 (day before the start of microdialysis) until after microdialysis and laser Doppler fluximetry had been finished on day 1. Healthy volunteers were discharged in the evening of day 1 and returned for a follow-up visit on the morning of day 2. Patients with T2DM were discharged on the morning of day 2. All participants returned for a follow-up visit 7 to 10 days after check-out.

Microdialysis for evaluation of VEGF-A protein production – procedure and endpoints

On day 0, six intradermal 50 µL injections of 0.9% saline were performed on the volar forearm at each of two sites, and two further sites were left untreated (Figure A1). The sites were assigned to be treated or untreated according to a randomization scheme. Approximately 20 minutes later, transversal microdialysis catheters with a 100 kDa cut-off were inserted ~10 mm intradermally, as in the subsequent phase 1 study. Microdialysis membranes were inserted in the middle of the six intradermal injections, or in the equivalent position at the untreated sites, on the volar side of the non-dominant forearm with ~2–5 cm between catheters. As in the phase 1 study, the catheters were connected to a 107 microdialysis pump (MDialysis, Sweden) and perfused with 0.9% saline at a rate of 0.5 µL/min.

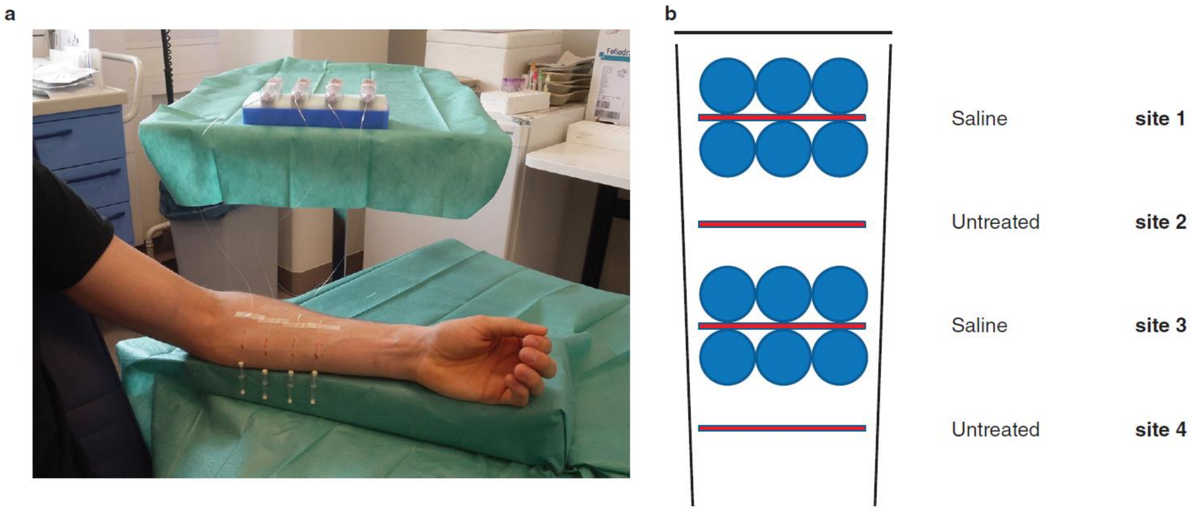

Figure A1. (a) Microdialysis set-up; (b) forearm application sites starting from the elbow flexure

Microdialysate sampling began immediately after catheter insertion, with dialysate collected at 0–2, 2–4 and 4–6 hours after insertion, and the catheters were then withdrawn. The following day, new catheters were inserted ~2–3 mm away from the previous ones, and dialysate was collected at 24–26 and 26–28 hours post-injection, as in the phase 1 study.

The primary endpoint of the microdialysis measurements was the concentration of VEGF-A protein in dialysate for each collection. As in the phase 1 study, measured VEGF-A protein levels were corrected for the dilution factor associated with sample processing.

Area under the curve (AUC) calculations were performed for the corrected VEGF-A concentrations in dialysate over two time periods: 0 to 6 hours post-injection (day 0) and 24 to 28 hours post-injection (day 1). The AUCs were calculated by summing the individual areas for each collection interval, where the individual area was given by the concentration for that interval multiplied by the time interval (i.e. 2 hours). The average concentration on day 0 and day 1 was obtained by dividing the relevant AUC by the time interval (i.e. 6 or 4 hours, respectively).

Acetylcholine iontophoresis with laser Doppler fluximetry – procedure and endpoints
On day 1, two very superficial intradermal 50 µL injections of 0.9% saline were performed on the contralateral forearm, at least 4 cm apart from each other (center to center), at least 5 cm from the antecubital fossa and 5 cm from the wrist (Figure A2). Two further sites were used as non-injection control sites according to a randomization list. Skin blood flow at the four injection sites approximately 4 hours post-injection was measured using the same laser

Doppler fluximetry with acetylcholine iontophoresis protocol as the subsequent phase 1 study (see main text Methods section).

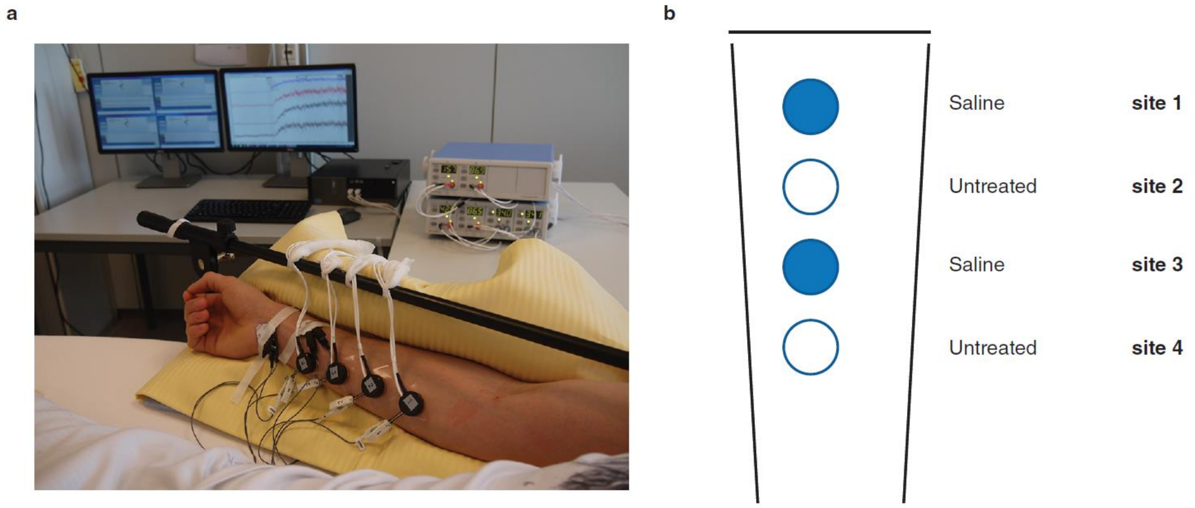

Figure A2. (a) Iontophoresis set-up; (b) forearm application sites starting from the elbow flexure

The following endpoints were determined using either Periflux software-captured parameters or visual inspection of the individual measured laser Doppler channels by an observer blinded to individual and treatment site:

- Basal (i.e. baseline) flux, measured before acetylcholine iontophoresis (perfusion units [PU]) – visual inspection
- Maximum flux, measured after acetylcholine iontophoresis (PU) – maximum single value calculated by software
- t_{lag}, time from start of iontophoresis to increase of flux (sec) – visual inspection
- $T_{0\text{–}plateau}$, time from start of iontophoresis to reach plateau (sec) – visual inspection
- $AUEC_{0\text{–}t}$, where time zero is the start of the iontophoresis and time ‘t’ is the end of the iontophoresis (PU*sec) – calculated by software
- $AUEC_{0\text{–}t,\ baseline\ corrected}$, where time zero is the start of the iontophoresis and time ‘t’ is the end of the iontophoresis (PU*sec) – calculated by software, depending on baseline
- $AUEC_{0\text{–}plateau}$ (PU*sec) – calculated by software, but $T_{0\text{–}plateau}$ determined by visual inspection

RESULTS AND CONCLUSIONS

Participants

Eight healthy volunteers and eight patients with T2DM, all white men, participated in this study. The median (range) age was 25 (20–60) years for the healthy volunteers and 57 (49–60) years for the patients with T2DM. Median (range) body mass index was 22.3 (20.9–26.3) kg/m^2 for the healthy volunteers and 30.2 (27.1–34.5) kg/m^2 for the patients with T2DM.

Safety

Overall, both the iontophoresis and microdialysis treatments were well tolerated by all participants, and no safety concerns were raised. Only two participants, both patients with T2DM, experienced AEs – one of mild musculoskeletal pain and one of moderate pain in an extremity. Both AEs were considered to be related to the study procedures. Mild redness and bruising were reported during the physical evaluations of local skin reactions.

VEGF-A in microdialysate

VEGF-A protein concentrations in microdialysate were similar in both healthy volunteers and patients with T2DM. Mean VEGF-A protein concentrations in microdialysate from all collection intervals ranged from 156–195 pg/mL in healthy volunteers, and from 139–193 pg/mL in patients with T2DM (Tables A1, A2, A3). There were no apparent differences between untreated and saline-treated sites, indicating no effect of saline treatment (Tables A1, A2, A3).

The VEGF-A protein measurements in microdialysate fluid were repeatable, with good agreement between replicate measurements for untreated and saline-treated sites. Inter-individual variability of VEGF-A concentration in patients with T2DM, as measured by coefficient of variation, was below 25% for all collection intervals across both untreated and saline-treated sites, being generally lower in the treated sites (Tables A2, A3).

Table A1. Concentrations of VEGF-A in microdialysate from healthy volunteers

**Treatment**	**Collection interval, day 0**			**Collection interval, day 1**	
	**0–2 h**	**2–4 h**	**4–6 h**	**24–26 h**	**26–28 h**
Untreated sites, n	8	8	8	7	8
Mean (SD), pg/mL	155.8 (32.8)	195.2 (30.2)	192.9 (31.9)	169.5 (32.8)	171.3 (36.6)
CV, %	21.1	15.5	16.5	19.3	21.3
Saline-treated sites, n	8	8	8	8	8
Mean (SD), pg/mL	170.9 (57.9)	178.0 (67.1)	173.3 (55.4)	194.4 (53.6)	190.4 (90.9)
CV, %	33.9	37.7	32.0	27.6	47.8

CV, coefficient of variation; SD, standard deviation; VEGF-A, vascular endothelial growth factor A.

Table A2. Concentrations of VEGF-A in microdialysate from patients with T2DM

**Treatment**	**Collection interval, day 0**			**Collection interval, day 1**	
	**0–2 h**	**2–4 h**	**4–6 h**	**24–26 h**	**26–28 h**
Untreated sites, n	8	8	8	8	8
Mean (SD), pg/mL	141.8 (22.7)	173.0 (17.3)	176.3 (15.6)	164.6 (33.7)	193.3 (47.7)
CV, %	16.0	10.0	8.8	20.5	24.7
Saline-treated sites, n	8	8	8	8	8
Mean (SD), pg/mL	138.6 (20.8)	180.6 (31.2)	171.3 (27.2)	142.4 (20.6)	178.2 (26.4)
CV, %	15.0	17.3	15.9	14.5	14.8

CV, coefficient of variation; SD, standard deviation; T2DM, type 2 diabetes mellitus; VEGF-A, vascular endothelial growth factor A.

Table A3. Average concentration of VEGF-A in microdialysate

**Treatment**	**Healthy individuals**		**Patients with T2DM**	
	**0–6 h**	**24–28 h**	**0–6 h**	**24–28 h**
Untreated sites, n	8	7	8	8
Mean (SD), pg/mL	181.3 (29.6)	171.9 (30.2)	163.7 (13.4)	179.0 (34.9)
CV, %	16.3	17.6	8.2	19.5
Saline-treated sites, n	8	8	8	8
Mean (SD), pg/mL	174.1 (55.7)	192.4 (50.5)	163.5 (23.7)	160.3 (23.2)
CV, %	32.0	26.2	14.5	14.5

CV, coefficient of variation; SD, standard deviation; T2DM, type 2 diabetes mellitus; VEGF-A, vascular endothelial growth factor A.

An analysis of variance was performed to evaluate intra-individual variability in the microdialysis data (Table A4). Intra-individual variability of average VEGF-A concentrations (at 0–6 hours and 24–28 hours post-injection) within both the pair of saline-treated sites and the pair of untreated sites was lower in patients with T2DM than in healthy volunteers and was similar for the untreated and saline-treated sites.

Table A4. Statistical analysis of intra-individual variability for average VEGF-A concentrations in microdialysate

Treatment	Healthy individuals		Patients with T2DM	
	**0–6 h**	**24–28 h**	**0–6 h**	**24–28 h**
Untreated sites, n	8	7	8	8
Intra-individual SD, pg/mL	25.5	29.0	18.0	22.6
Saline-treated sites, n	8	8	8	8
Intra-individual SD, pg/mL	43.5	70.4	21.9	24.2

Data are from a repeated measures ANOVA model, with endpoint as the dependent variable, repetition number as a fixed effect and individual as the blocking variable for the repeated measures.

ANOVA, analysis of variance; SD, standard deviation; T2DM, type 2 diabetes mellitus; VEGF-A, vascular endothelial growth factor A.

Microdialysis for assessment of VEGF-A concentrations in forearm skin was concluded to be feasible, safe and well tolerated in healthy volunteers and patients with T2DM, supporting use of the technique in the phase 1 AZD8601 study.

Skin blood flow by laser Doppler fluximetry

Patients with T2DM had lower values for all seven fluximetry endpoints, at both untreated and saline-treated sites, than healthy volunteers, reflecting the impaired microcirculation in these patients (Tables A5, A6). Mean basal flux at saline-treated sites was 17.17 PU in patients with T2DM and 31.33 PU in healthy volunteers.

Saline injection had the effect of increasing basal flux in both groups of volunteers, the expected physiological response of increased blood flow in the injured region (Tables A5, A6); mean basal flux in patients with T2DM was 17.17 PU at saline-treated sites versus 9.13 PU at untreated sites (Table, A6). $AUEC_{0–t}$ was higher at saline-treated sites in healthy volunteers but not in T2DM patients (Tables A5, A6).

Inter-individual variability for the fluximetry endpoints, as measured using coefficient of variation, was 30% or above for the majority of the seven endpoints at all sites in both healthy volunteers and patients with T2DM (Tables A5, A6). In patients with T2DM, inter-individual variability was lower for the saline-treated sites versus untreated sites for most endpoints, being lowest for maximum flux, t_{lag}, and $AUEC_{0–t}$ (Table A6).

Table A5. Laser Doppler fluximetry endpoints in healthy volunteers

**Parameter**	**Untreated sites (n = 8)**	**Saline-treated sites (n = 8)**
Basal flux		
Mean (SD), PU	12.76 (6.59)	31.33 (18.06)
CV, %	51.6	57.7
Maximum flux		
Mean (SD), PU	111.2 (33.47)	127.6 (48.79)
CV, %	30.1	38.2
t_{lag}		
Mean (SD), sec	48.5 (41.4)	31.0 (17.1)
CV, %	85.3	55.1
$t_{0\text{–plateau}}$		
Mean (SD), sec	348.3 (110.8)	242.5 (102.5)
CV, %	31.8	42.3
$AUEC_{0\text{–}t}$		
Mean (SD), PU*sec	38152 (9153)	48391 (16362)
CV, %	24.0	33.8
$AUEC_{0\text{–}t\text{, baseline corrected}}$		
Mean (SD), PU*sec	30515 (9233)	28792 (17673)
CV, %	30.3	61.4
$AUEC_{0\text{–plateau}}$		
Mean (SD), PU*sec	17758 (6254)	15427 (6396)
CV, %	35.2	41.5

$AUEC_{0\text{–plateau}}$, area under the effect curve from start of acetylcholine iontophoresis to reach plateau; $AUEC_{0\text{–}t}$, area under the effect curve from start to end; CV, coefficient of variation; PU, perfusion units; SD, standard deviation; $t_{0\text{–plateau}}$, time from start of iontophoresis to reach plateau; t_{lag}, time from start of iontophoresis to increase of flux.

Table A6. Laser Doppler fluximetry endpoints in patients with T2DM

**Parameter**	**Untreated sites (n = 8)**	**Saline-treated sites (n = 8)**
Basal flux		
Mean (SD), PU	9.13 (2.91)	17.17 (8.18)
CV, %	31.9	47.7
Maximum flux		
Mean (SD), PU	70.77 (27.01)	61.22 (16.59)
CV, %	38.2	27.1
t_{lag}		
Mean (SD), sec	38.3 (18.2)	21.8 (7.0)
CV, %	47.7	32.1
$t_{0–plateau}$		
Mean (SD), sec	295.1 (106.3)	223.4 (92.8)
CV, %	36.0	41.5
$AUEC_{0–t}$		
Mean (SD), PU*sec	24981 (11636)	23824 (7421)
CV, %	46.6	31.2
$AUEC_{0–t,\ baseline\ corrected}$		
Mean (SD), PU*sec	19204 (9850)	13606 (5954)
CV, %	51.3	43.8
$AUEC_{0–plateau}$		
Mean (SD), PU*sec	9571 (5600)	6972 (3009)
CV, %	58.5	43.2

$AUEC_{0–plateau}$, area under the effect curve from start of acetylcholine iontophoresis to reach plateau; $AUEC_{0–t}$, area under the effect curve from start to end; CV, coefficient of variation; PU, perfusion units; SD, standard deviation; $t_{0–plateau}$, time from start of iontophoresis to reach plateau; t_{lag}, time from start of iontophoresis to increase of flux.

A repeated measures analysis of variance was performed to evaluate intra-individual variability of the fluximetry endpoints (Table A7), which was found to be generally lower for patients with T2DM than for healthy volunteers. In patients with T2DM, intra-individual variability was lower for the pair of saline-treated sites versus the pair of untreated sites for maximum flux, $T_{0–plateau}$, $AUEC_{0–t}$, $AUEC_{0–t,\ baseline\ corrected}$ and $AUEC_{0–plateau}$, similar for t_{lag}, and higher for basal flux.

Table A7. Statistical analysis of intra-individual variability for Laser Doppler fluximetry endpoints

Parameter	Intra-individual SD			
	**Healthy individuals**		**Patients with T2DM**	
	**Untreated sites (n = 8)**	**Saline-treated sites (n = 8)**	**Untreated sites (n = 8)**	**Saline-treated sites (n = 8)**
Basal flux, PU	7.44	12.18	2.59	11.31
Maximum flux, PU	54.15	66.50	32.99	23.03
t_{lag}, sec	34.72	10.14	8.602	8.854
$t_{0\text{–plateau}}$, sec	105.6	107.9	111.9	83.53
$AUEC_{0\text{–}t}$, PU*sec	15514	21954	15372	8898
$AUEC_{0\text{–}t\text{, baseline corrected}}$, PU*sec	16126	20736	13994	6972
$AUEC_{0\text{–plateau}}$, PU*sec	8848	8256	9274	4198

Data are from a repeated measures analysis of variance model, with parameter as the dependent variable, repetition number as a fixed effect and individual as the blocking variable for the repeated measurements.

$AUEC_{0\text{–plateau}}$, area under the effect curve from start of acetylcholine iontophoresis to reach plateau; $AUEC_{0\text{–}t}$, area under the effect curve from start to end; CV, coefficient of variation; PU, perfusion units; SD, standard deviation; $t_{0\text{–plateau}}$, time from start of iontophoresis to reach plateau; T2DM, type 2 diabetes mellitus; t_{lag}, time from start of iontophoresis to increase of flux.

Acetylcholine iontophoresis with laser Doppler fluximetry was concluded to be a feasible and safe method for studying differences in physiology and pathophysiology of forearm skin microcirculation in healthy volunteers and patients with T2DM, supporting use of the technique in the phase 1 AZD8601 study.

Basal flux, maximum flux and $AUEC_{0\text{–t}}$ were concluded to be the most suitable endpoints for the phase 1 study. Maximum flux and $AUEC_{0\text{–t}}$ had the smallest inter-individual variability in saline-treated sites for both healthy volunteers and patients with T2DM; these parameters were calculated by the laser Doppler fluximetry software so also had the benefit of being observer-independent. Basal and maximum flux, and $AUEC_{0\text{–t}}$, were also considered the most suitable endpoints for assessing the effect of acetylcholine-induced vasodilation.

Supplementary Figures

Supplementary Figure 1. Injection sites

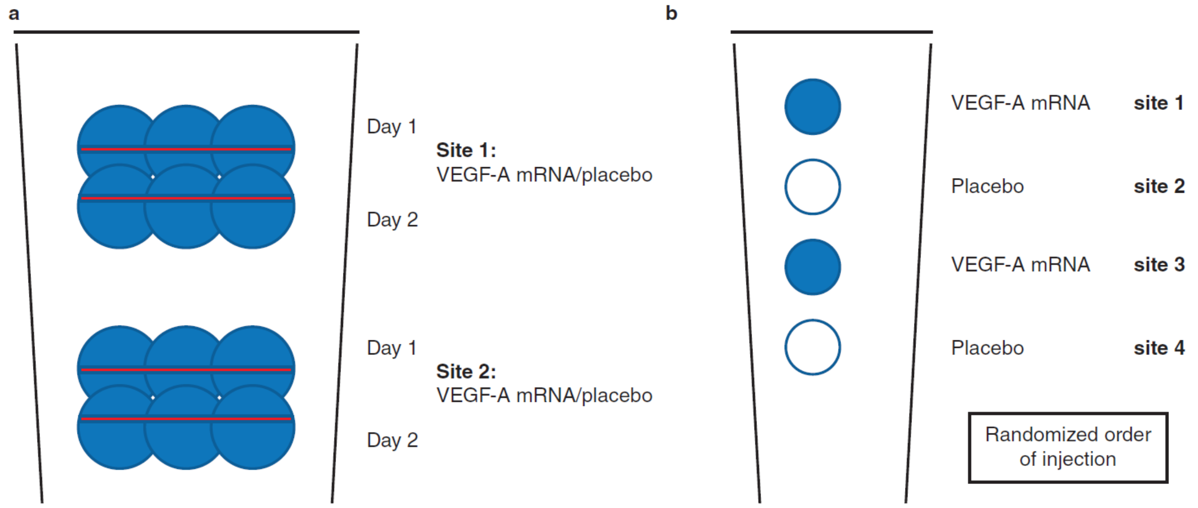

(a) Study Part A. Participants in part A were randomized 1:1:1 to receive VEGF-A mRNA at site 1 and placebo at site 2, placebo at site 1 and VEGF-A mRNA at site 2 or placebo at both sites, given as six 50 µL intradermal injections per site in the pattern shown. After 60–90 minutes, a microdialysis membrane was inserted at each site as shown (red line), for collection of microdialysate 1.5–7.5 hours after administration, and the membranes were withdrawn. The following day (23.5–24.0 h after administration), a new microdialysis membrane was inserted 3–5 mm from the previous insertion location at each site as shown, for collection of microdialysate 24–28 hours after administration.

(b) Study Part B. Participants in part B received VEGF-A mRNA or placebo at each of injection sites 1, 2, 3 and 4 in a randomized order, given as a single 50 µL injection at each site.

VEGF-A mRNA, vascular endothelial growth factor A modified messenger RNA.

Supplementary Figure 2. Correlations of local skin blood flow with VEGF-A levels

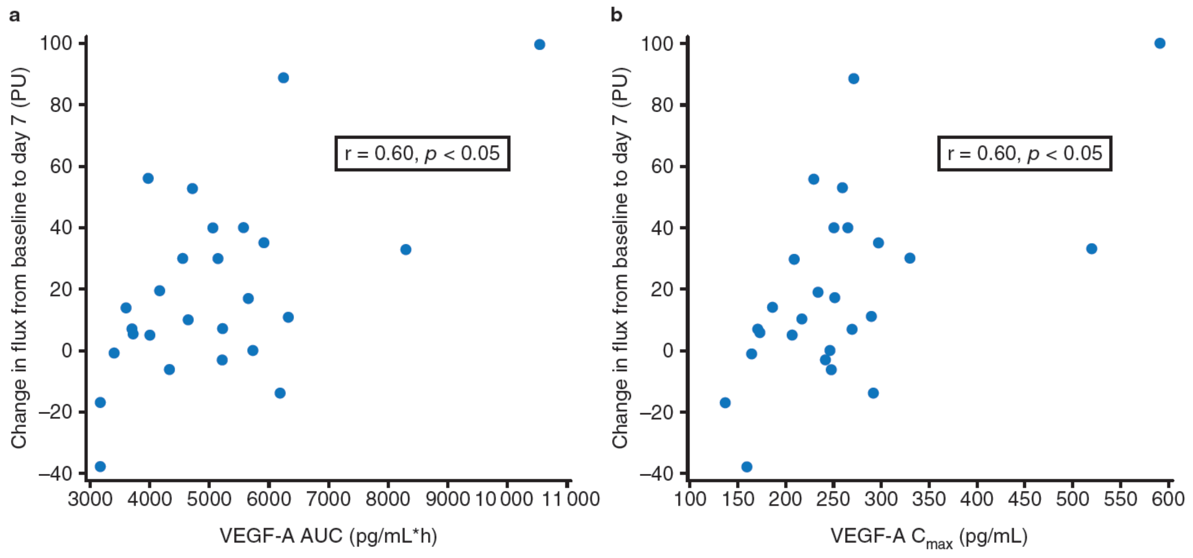

Post hoc Pearson correlation between change in local skin blood flow and (a) total or (b) maximal local microdialysate VEGF-A protein concentration in Study Part A. Nominal *p* values.

VEGF-A AUC, area under the curve of VEGF-A protein concentration in microdialysate between 3.5 and 28 h after administration; VEGF-A C_{max}, maximum VEGF-A protein concentration in microdialysate between 3.5 and 28 h; VEGF-A, vascular endothelial growth factor A.

Supplementary Figure 3. VEGF-A mRNA characteristics

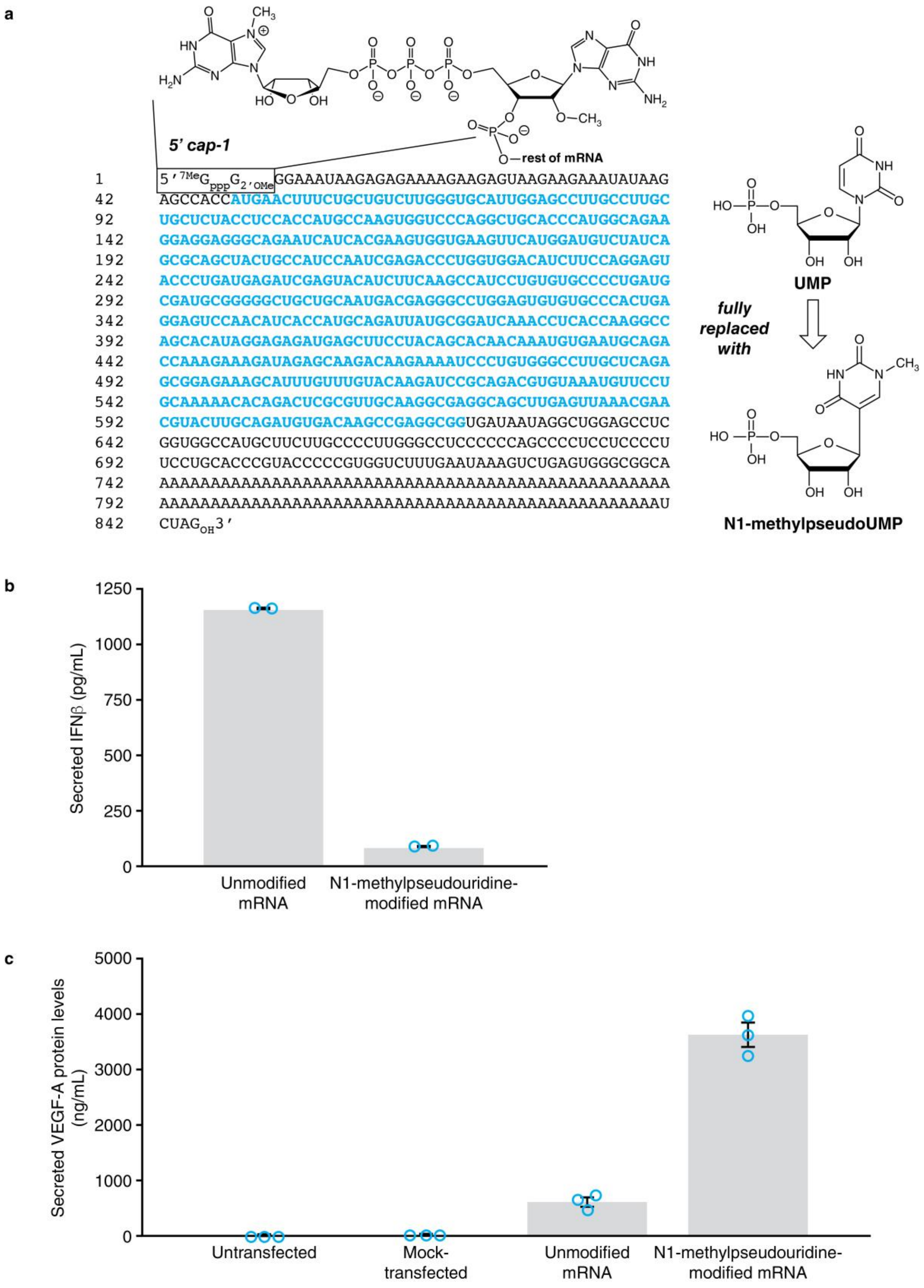

(a) Nucleotide sequence and chemical structure of the VEGF-A mRNA drug substance. Blue font indicates VEGF-A_{165} protein-coding sequence.

(b) Interferon-β induction in BJ fibroblasts by modified and unmodified mRNA. mRNAs encoding nanoluciferase and with the indicated modifications were synthesized *in vitro.* BJ fibroblasts (CRL-2522, American Type Culture Collection) cultured in 96-well plates were transfected with 250 ng mRNA complexed with 0.5 µL Lipofectamine 2000 (Fisher). Supernatants were collected 48 h later and interferon-β protein levels were quantified by ELISA (41410-1, PBL Assay Science). Individual data points (n = 2), mean and SD are shown.

(c) Expression of VEGF-A from HeLa cells transfected with modified and unmodified VEGF-A mRNA. mRNAs encoding VEGF-A and with the indicated modifications were synthesized *in vitro* and purified using MEGAclear kits (Ambion). Sub-confluent HeLa cells (CCL-2, American Type Culture Collection) cultured in 96-well plates were transfected with 250 ng mRNA complexed with 0.4 µL Lipofectamine 2000 (Fisher). Supernatants were collected the following day and VEGF-A protein levels were quantified by ELISA (DVE00, R&D Systems). Individual data points (n = 3), mean and SD are shown.

A, adenine; C, cytosine; ELISA, enzyme-linked immunosorbent assay; G, guanine; Me, methyl; p, inorganic phosphate; SD, standard deviation; U, N1-methylpseudouridine.

Supplementary Tables

Supplementary Table 1. Study stopping criteria

Adverse events

- ≥ 1 participant receiving VEGF-A mRNA reporting a serious or intolerable adverse event
- ≥ 2 participants with severe injection-site reactions related to VEGF-A mRNA

Cardiovascular findings

- ≥ 2 participants receiving VEGF-A mRNA with any of the following:
  - QTcF > 500 ms or increase in QTcF > 60 ms from baseline for > 5 minutes
  - Symptomatic resting supine heart rate < 45 bpm
  - Asymptomatic resting supine heart rate < 40 bpm for ≥ 10 minutes while awake
  - Resting supine heart rate > 125 bpm for ≥ 10 minutes
  - Increase in systolic BP of > 40 mmHg to > 180 mmHg for ≥ 10 minutes
  - Asymptomatic decrease in systolic BP of > 30 to < 70 mmHg for ≥ 10 minutes
  - Symptomatic decrease in systolic BP of > 30 mmHg (excluding vasovagal reaction)

Laboratory findings

- ≥ 1 participant receiving VEGF-A with an unexplained increase in AST or ALT to ≥ 3 × ULN and total bilirubin ≥ 2 × ULN
- ≥ 2 participants receiving VEGF-A mRNA with any of the following:
  - AST or AST ≥ 3 × ULN
  - Bilirubin or ALP ≥ 2 × ULN
  - Leukocyte count < 2.0 × 10^9/L
  - Neutrophil count < 1.0 × 10^9/L
  - Platelet count < 75 × 10^9/L
  - Serum creatinine increase to ≥ 1.5 × ULN

BP, blood pressure; ALP, alkaline phosphatase; ALT, alanine aminotransferase; AST, aspartate aminotransferase; QTcF, QT interval corrected for heart rate using Fridericia’s formula; ULN, upper limit of normal; VEGF-A mRNA, vascular endothelial growth factor A modified messenger RNA.

Supplementary Table 2. Numbers of participants whose laboratory values shifted to abnormally low or high values from other baseline values

**Parameter**	**Part A (n = 27)**		**Part B (n = 15)**
	Placebo only (n = 9)	VEGF-A mRNA/ placebo (n = 18)	VEGF-A mRNA/ placebo (n = 15)
Alanine aminotransferase, n_{low}, n_{high}	0, 0	0, 2	0, 1
Albumin, n_{low}, n_{high}	0, 0	1, 0	0, 0
Alkaline phosphatase, n_{low}, n_{high}	0, 0	0, 0	0, 0
Aspartate aminotransferase, n_{low}, n_{high}	0, 0	0, 1	0, 1
Bilirubin, n_{low}, n_{high}	0, 0	0, 1	0, 0
C-reactive protein, n_{low}, n_{high}	0, 1	0, 3	0, 1
Calcium, n_{low}, n_{high}	1, 0	0, 0	1, 0
Creatinine, n_{low}, n_{high}	0, 0	2, 0	1, 0
Direct bilirubin, n_{low}, n_{high}	0, 0	0, 1	0, 0
Gamma glutamyl transferase, n_{low}, n_{high}	0, 1	0, 1	0, 0
Glucose, n_{low}, n_{high}	0, 0	0, 2	0, 0
Indirect bilirubin, n_{low}, n_{high}	0, 0	0, 0	0, 0
Phosphate, n_{low}, n_{high}	0, 0	1, 0	1, 1
Potassium, n_{low}, n_{high}	0, 0	0, 0	1, 1
Sodium, n_{low}, n_{high}	5, 0	5, 0	1, 0
Urea nitrogen, n_{low}, n_{high}	0, 1	1, 3	0, 1
Basophils, n_{low}, n_{high}	0, 1	0, 1	0, 0
Eosinophils, n_{low}, n_{high}	0, 0	0, 0	0, 0
Erythrocyte mean corpuscular hemoglobin concentration, n_{low}, n_{high}	0, 0	3, 0	0, 2
Erythrocyte mean corpuscular hemoglobin, n_{low}, n_{high}	0, 2	0, 2	0, 1
Erythrocyte mean corpuscular volume, n_{low}, n_{high}	0, 0	0, 0	0, 0
Erythrocytes, n_{low}, n_{high}	1, 0	1, 1	0, 0
Haematocrit, n_{low}, n_{high}	0, 0	5, 0	0, 0
Hemoglobin, n_{low}, n_{high}	0, 0	1, 0	0, 1
Leukocytes, n_{low}, n_{high}	0, 0	0, 1	0, 2
Lymphocytes, n_{low}, n_{high}	0, 0	1, 0	0, 0
Monocytes, n_{low}, n_{high}	0, 0	1, 0	4, 0
Neutrophils, n_{low}, n_{high}	0, 0	0, 0	0, 3
Platelets, n_{low}, n_{high}	0, 0	3, 0	0, 0
Reticulocytes, n_{low}, n_{high}	0, 2	0, 2	0, 0
Activated partial thromboplastin time, n_{low}, n_{high}	1, 0	2, 0	0, 0
Prothrombin international normalized ratio, n_{low}, n_{high}	0, 0	0, 0	0, 0

Shifts to abnormally high values are shifts from normal or abnormally low baseline values; shifts to abnormally low values are shifts from normal or abnormally high baseline values. Shifts to abnormally high values are based on maximum values after administration; shifts to abnormally low values are

based on minimum values after administration.

VEGF-A mRNA, vascular endothelial growth factor A modified messenger RNA.

Supplementary Table 3. Participants with at least one abnormal vital sign reading during the study

Abnormal vital sign	Part A (n = 27)[a]		Part B (n = 15)[b]
	Placebo only (n = 9)	VEGF-A mRNA/ placebo (n = 18)	VEGF-A mRNA/ placebo (n = 15)
Systolic BP < 90 mmHg, n (%)	0 (0)	1 (5.6)	0 (0)
Diastolic BP < 45 mmHg, n (%)	0 (0)	0 (0)	0 (0)
Pulse < 45 bpm, n (%)	0 (0)	0 (0)	0 (0)
Systolic BP > 140 mmHg, n (%)	7 (77.8)	13 (72.2)	8 (53.3)
Diastolic BP > 90 mmHg, n (%)	1 (11.1)	6 (33.3)	5 (33.3)
Pulse > 90 bpm. n (%)	2 (22.2)	1 (5.6)	0 (0)

[a]BP readings were taken at screening and on day −1, day 0 pre-treatment and at 5, 20 and 30 minutes and 1, 2, 3, 4, 6, 8, 12, 24, 36 and 48 hours post-treatment (and at 72, 96, 120 and 144 hours post-treatment for participants leaving the study center on day 7), day 7, day 14, and at the monthly follow-up visits.

[b]BP readings were taken at screening and on day −1, day 0 pre-treatment and at 30 minutes and 1, 2, 3, 4, 6, 8, 12 and 24 hours post-treatment, day 14, and at the monthly follow-up visits.

BP, blood pressure; VEGF-A mRNA, vascular endothelial growth factor A modified messenger RNA.
