# Supplementary material for: Intradermal delivery of modified mRNA encoding VEGF-A in patients with type 2 diabetes
Source: Nat Commun. 2019 Feb 20;10:871. doi: 10.1038/s41467-019-08852-4 (PMC6382754; doi:10.1038/s41467-019-08852-4)
Supplement: Supplementary file 5 — Description of Additional Supplementary Files [file 41467_2019_8852_MOESM5_ESM.pdf]

Supplementary Dataset 1

Largest mean changes in laboratory parameters from baseline.
