# Supplementary material for: Intradermal delivery of modified mRNA encoding VEGF-A in patients with type 2 diabetes
Source: Nat Commun. 2019 Feb 20;10:871. doi: 10.1038/s41467-019-08852-4 (PMC6382754; doi:10.1038/s41467-019-08852-4)
Supplement: Supplementary file 3 — Peer Review File [file 41467_2019_8852_MOESM3_ESM.pdf]

Editorial Note: this manuscript has been previously reviewed at another journal that is not operating a transparent peer review scheme. This document only contains reviewer comments and rebuttal letters for versions considered at Nature Communications.
